# Low-Temperature, Efficient Synthesis of Highly Crystalline Urchin-like Tantalum Diboride Nanoflowers

**DOI:** 10.3390/ma15082799

**Published:** 2022-04-11

**Authors:** Delei Liu, Jianghao Liu, Peikan Ye, Haijun Zhang, Shaowei Zhang

**Affiliations:** 1 The State Key Laboratory of Refractories and Metallurgy, Wuhan University of Science and Technology, Wuhan 430081, China; 15927529267@163.com (D.L.); yepk2022@126.com (P.Y.); 2 College of Engineering, Mathematics and Physical Sciences, University of Exeter, Exeter EX4 4QF, UK; s.zhang@exeter.ac.uk

**Keywords:** ultra-high-temperature ceramics, tantalum diboride, microwave, molten-salt synthesis, single crystal, urchin-like, nanoflower, toughening

## Abstract

Urchin-like tantalum diboride (TaB_2_) nanoflowers were successfully synthesized via a high-efficiency and energy-saving methodology, molten-salt and microwave co-modified boro/carbothermal reduction, using less expensive B_4_C as a reducing agent. By taking advantage of the synergistic effects of the molten-salt medium and microwave heating conditions, the onset formation temperature of TaB_2_ was drastically reduced to below 1000 °C, and phase-pure powders of TaB_2_ nanoflowers were obtained at temperatures as low as 1200 °C within only 20 min. Notably, the present temperature conditions were remarkably milder than those (>1500 °C for several hours) required by conventional reduction methods, which use the strong, but expensive, reducing agent, elemental boron. The resulting urchin-like TaB_2_ nanoflowers consisted of numerous uniform single-crystalline nanowires with lengths up to 4.16 μm, and high aspect ratios >10. This result indicated that the as-synthesized urchin-like TaB_2_ nanoflowers possessed high specific surface area and anisotropic morphology, which were favorable not only for sintering, but also for toughening their bulk counterparts.

## 1. Introduction

As one of the most important ultra-high-temperature ceramics (UHTCs) for potential applications at >2000 °C, tantalum diboride (TaB_2_) has attracted extensive interest in recent decades due to its rapidly rising demand for high-end structural applications in harsh environments, such as the propulsion systems in new-generation space vehicles, combustion chambers of rockets, and the leading edge of reentry hypersonic missiles [1,2,3]. Under such extreme operating conditions, the frequent occurrence of sharp heating–cooling cycles results in high thermal stress in TaB_2_-based protective materials, greatly challenging their thermal shock resistance as well as the relevant main factor, fracture toughness [4,5]. Thus, it is of significant importance to prepare monolithic TaB_2_ with sufficient fracture toughness.

To overcome the poor toughness of many ceramics, the introduction of a secondary reinforcement phase with an anisotropic (e.g., rod- or plate-like) morphology into the ceramic matrix for toughening matrix materials has been demonstrated to be effective [6,7]. Nevertheless, due to the ultra-high service temperature, the number of the second-phase candidates applicable to TaB_2_-based materials was extremely limited. Considering this, “self-toughening” was regarded as the only feasible way to enhance the fracture toughness of TaB_2_-based materials [8]. Thus, their powder counterparts, consisting of highly crystalline anisotropy-shaped TaB_2_ particles, were perceived to be desirable for preparation of high-performance TaB_2_-based materials [9,10,11,12]. 

Unfortunately, with the boro/carbothermal reduction methodology conventionally utilized for the industrial preparation of TaB_2_ and other raw powders of UHTCs as well as TaB_2_, it was still a challenging task to endow the powder product with anisotropic morphology and the resulting potential for toughness enhancement [13,14]. Additionally, the preparation of TaB_2_ powders via conventional thermal reduction commonly necessitates severe temperature conditions, including high firing temperature (>1500 °C) and long dwelling time (dozens of hours), which not only dramatically increases corresponding energy and time consumptions, but also seriously deteriorates the sinterability of the powder product. For instance, Guo et al. synthesized TaB_2_ with amorphous morphology using the conventional borothermal reduction (BTR) at temperatures as high as 1550 °C for one hour, by using highly expensive elemental boron as both B source and reducing agent [13]. Zhang et al. successfully prepared TaB_2_ powders by heating the raw powders of Ta_2_O_5_, B_4_C and graphite at 1600 °C, albeit both needle-like and rounded particles coexisted in the final product [15]. In summation, the current thermal-reduction-based methodology for mass preparation of TaB_2_ powders suffers from the drawbacks of high preparation cost and poor quality of products, which are mainly attributable to the intrinsically low diffusion coefficient of TaB_2_ and the resulted low rates of both synthetic reaction and crystal growth [16].

In this view, the prospect of introducing a liquid phase was deemed promising for improving the diffusion rate of TaB_2_, and therefore enhancing its synthesis and epitaxial crystal growth. For instance, Ren et al. exploited low-melting-point Ta-B-C-O precursors to supply the boro/carbothermal reduction reaction for synthesizing TaB_2_ with liquid medium. However, no formation of anisotropic TaB_2_ was not reported despite the requirement of a high processing temperature of 1500 °C was still demanded, which was considered to be mainly due to the decomposition loss of the low-melting-point organic-based medium, before the temperature desirable for crystal growth of TaB_2_ was attained [17]. As a result, inorganic salt with a relatively higher melting point was regarded as a promising medium for solid-state synthetic reaction of high-temperature materials, including TaB_2_. For instance, Ran et al. utilized the molten-salt-assisted BTR method to prepare rod-like TaB_2_ powders after a one-hour dwelling period at a reduced temperature of 1000 °C, albeit the requirement of a large amount of boron powders resulted in high material costs [18].

In addition, microwaves have also been verified to be capable of effectively accelerating not only synthetic reactions, but also crystal growth of high-temperature materials. Moreover, it was attainable for the microwave field to cooperate with the molten-salt field, therefore jointly enhancing the thermodynamic favorability of synthetic reactions and facilitating self-assembly of anisotropic-shaped crystals [19,20]. Accordingly, in our previous research, microwaves were utilized to further modify the molten-salt-assisted BCTR method to prepare ZrB_2_ and ZrB_2_-SiC powders with anisotropic morphologies under much milder conditions [21,22].

Consequently, in this work, molten-salt and microwave comodified boro/carbothermal reduction was developed for synthesizing TaB_2_, using Ta_2_O_5_ and B_4_C as raw materials. The effects of various important parameters on the synthesis of TaB_2_ were elaborately regulated to save energy and efficiently prepare single-phase TaB_2_ powders with high specific surface area and anisotropic morphology.

## 2. Materials and Methods

### 2.1. Raw Materials

The raw materials of tantalum oxide (Ta_2_O_5_; purity > 98.5%; average particle size of 0.39 μm; Shanghai Aladdin Bio-Chem Technology Co., Ltd., Tianjin, China) and boron carbide (B_4_C; purity > 98.0%; average particle size of 3.74 μm; Mudanjiang Jingangzuan Boron-Carbide Co., Ltd., Mudanjiang, China) were used as received for synthesis of TaB_2_. Salts of sodium chloride and potassium chloride (NaCl and KCl; purities > 99.9%; Bodi Chem. Co., Ltd., Tianjin, China) were used as a reacting medium. All above reagents were directly used without further purification or modification.

7Ta_2_O_5_(s) + 11B_4_C(s) = 14TaB_2_(s) + 8B_2_O_3_(l) + 11CO(g) (1)

For compensating for the volatilization loss of boron species during the synthetic reaction of TaB_2_, certain excess amounts of B_4_C were added into the raw material powders (according to reaction (1)). For the reacting medium, the molar ratio between NaCl and KCl was fixed at 1:1. The batch compositions and processing parameters for the molten-salt and microwave-assisted boro/carbothermal reduction (MSM-BCTR) and the conventional BCTR are listed in Table 1. The corresponding products are labeled as MSMBC-(1–7).

### 2.2. Experiment Procedure

Similar to a typical MSM-BCTR process for synthesizing TaB_2_, the reactants, consisting of Ta_2_O_5_/B_4_C and the salt mixture of NaCl/KCl in a weight ratio of 1:2, were initially mixed in a corundum crucible. Then, the crucible, contained by a SiC saggar, was positioned in the heating area of a microwave furnace (HAMiLab-V3000; 3 kW; 2.45 GHz; Changsha Longtech Co., Ltd., Changsha, China). The space between the saggar and crucible was filled with green silicon carbide powders to facilitate the microwave heating of the raw material powders, and the operating temperature was monitored in real-time by an infrared thermometer. Afterwards, in a flowing argon atmosphere, the raw material powders were heated at a constant rate of 10 °C/min to the preset temperature range of 1000–1200 °C, then held for 0–20 min before cooling naturally to the ambient temperature. The resultant powders were ground and repeatedly rinsed with deionized water to completely remove the residual salt, followed by overnight drying in a vacuum oven at 80 °C, before undergoing the following characterization and testing.

### 2.3. Characterization and Testing

Phase compositions of the powder products were examined by X-ray diffraction (XRD, Empyrean, PANalytical, Almelo, The Netherlands) with CuK*α* radiation (*λ* = 0.1542 nm). The morphology and microstructure of as-obtained TaB_2_ powders were characterized using a field-emission scanning electron microscope (FE-SEM, Nova400NanoSEM, Philips, Amsterdam, The Netherlands) equipped with an energy dispersive spectrometer (EDS) and transmission electron microscope (TEM, JEM-2100UHRSTEM, JEOL, Tokyo, Japan), and their mean aspect ratio was estimated based on the measurements of at least 200 well-grown rod-like particles.

## 3. Results

**Figure 1:**
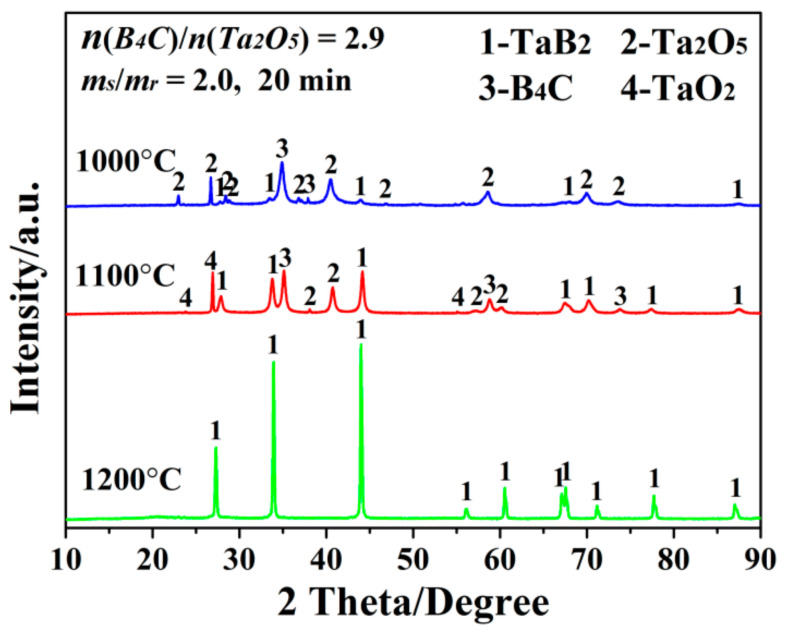
XRD patterns of the samples resultant from MSM-BCTR treatments at 1000–1200 °C/20 min, with the identical processing parameters of *n*(B_4_C)/*n*(Ta_2_O_5_) = 2.9 and *m_s_*/*m_r_* = 2.0.

As confirmed by our previous researchers [21,22], the present MSM-BCTR method was capable of greatly accelerating the BCTR reaction for synthesizing boron-based UHTCs at reduced temperatures. Therefore, the syntheses of TaB_2_ were performed at a relatively low temperature range of 1000–1200 °C, for an identical dwelling time of 20 min, with batch powder composition *n*(B_4_C)/*n*(Ta_2_O_5_) = 2.9, and weight ratio between salts and reactants of *m_s_*/*m_r_* = 2.0. Specifically, as the firing temperature reached 1000 °C (Figure 1), although there existed a lot of un-reacted Ta_2_O_5_ and B_4_C, as well as the intermediate product of TaO_2_ (produced by partial reduction of Ta_2_O_5_), in the final product (MSMBC-1), a minor amount of TaB_2_ could also be identified. This result indicated that the expected formation reaction of TaB_2_ (reaction (1)) was initiated at a temperature lower than 1000 °C, which was dramatically lower than that (up to 1550 °C) required by the conventional BCTR method using an identical type of reducing agent [9]. Subsequently, on increasing the temperature to 1100 °C (MSMBC-2), the intensities of the diffraction peaks belonging to TaB_2_ increased, while those of the residual reactants and intermediate product accordingly decreased, revealing the rising temperature had a positive effect on enhancing the synthesis of TaB_2_. More interestingly, upon further increasing temperature to 1200 °C, all observable diffraction peaks of the product (MSMBC-3) were indexed to TaB_2_, demonstrating that phase-pure TaB_2_ powders were successfully obtained. This result verified that, although the temperature was as low as 1200 °C, the synthetic reaction of TaB_2_ was still highly efficient, remarkably superior to that (at least one hour) in the conventional BCTR using the stronger reducing agent, boron [18].

**Figure 2:**
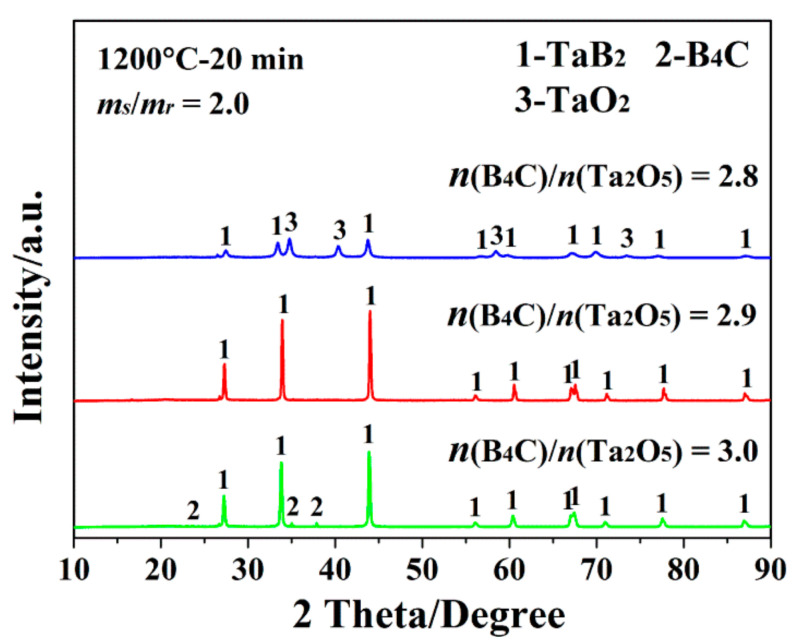
XRD patterns of the samples prepared by MSM-BCTR method at 1200 °C/20 min, with the identical addition amount of salt medium (*m_s_*/*m_r_* = 2.0) and the different molar ratios of B_4_C and Ta_2_O_5_ (*n*(B_4_C)/*n*(Ta_2_O_5_)) of 2.8–3.0.

Moreover, the additional amount of reducing agent was deemed another key factor that greatly influenced the synthetic result of TaB_2_, because insufficient dosages of reducing agent would have impeded the expected completion of the synthetic reaction, while excessive dosages would have induced carbon residue in the final product, seriously and irreversibly deteriorating its purity and sinterability. Given this, the following experiments were performed using different addition amounts of B_4_C in the original powder batches. On one hand, as shown in Figure 2, in sharp contrast with MSMBC-3 (composed of single-phase TaB_2_), the sample (MSMBC-4) prepared under identical processing conditions, but with a slightly less amount of B_4_C (with the ratio of *n*(Ta_2_O_5_)/*n*(B_4_C) decreased from 2.9 to 2.8), contained a trace amount of TaO_2_ but no B_4_C or C, indicating the expected synthetic reaction of TaB_2_ failed to reach completion due to the insufficient amount of reducing agent. On the other hand, upon increasing the ratio of *n*(Ta_2_O_5_)/*n*(B_4_C) to 3.0, there existed no oxygen-containing impurity but a minor amount of B_4_C remained in the product (MSMBC-5), implying the present addition amount of reducing agent was excessive. So, it was reasonable to conclude that, under the present MSM-BCTR conditions, the optimal molar ratio between Ta_2_O_5_ and B_4_C was approximately 2.9, which was favorable for the completion of the expected synthetic reaction, while minimizing the content of carbon-containing residue in the final TaB_2_ product powders.

**Figure 3:**
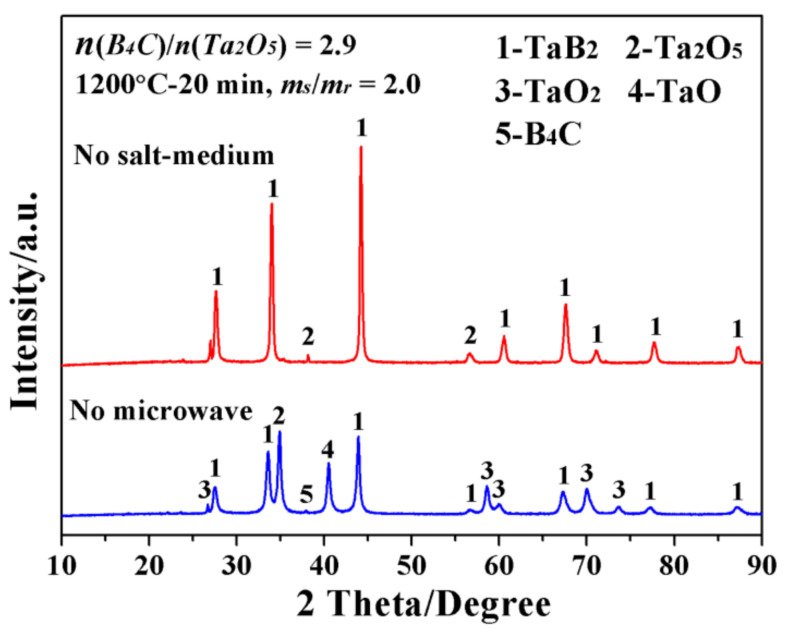
XRD patterns of the samples prepared by MSM-BCTR method under the optimized processing conditions while not using the conditions without using either microwave heating or molten-salt medium.

Such a prominent achievement of low-temperature and high efficiency preparation of single-phase TaB_2_ powders was regarded to be closely related to the specific MSM-BCTR conditions, characterized by microwave heating and salt medium. For the purpose of clarifying their independent impacts on the synthesis of TaB_2_, the following experiments were conducted under the optimized processing conditions, without either microwave heating or molten-salt medium, and then compared. On one hand, in the absence of salt medium (corresponding XRD patterns were presented by Figure 3), there existed a small amount of Ta_2_O_5_ in addition to TaB_2_ in the product (MSMBC-6), revealing the molten-salt medium had an obvious positive effect on accelerating the synthetic reaction of TaB_2_. On the other hand, in the case of conventional heating (without microwave), multiple types of oxygen-containing impurities (including Ta_2_O_5_, TaO_2_ and TaO), as well as unreacted B_4_C, remained in the powder product (MSMBC-7). It was worth noting that TaC, as a common byproduct of TaB_2_ synthesized by the conventional BCTR method, was not traced in this sample, although the corresponding synthetic reaction (reaction (2)) remained thermodynamically possible. Such seemingly contradicting results could be explained by the fact that, although reaction (2) prevailed to form TaC, it would be subsequently converted into TaB_2_ via reaction (3), which was concurrently enhanced by the molten-salt medium. Therefore, it is reasonable to consider that the combined effects of molten-salt medium and microwave heating had significantly combined effects on the synthesis of TaB_2_, including (1) accelerating the targeted reaction by promoting the diffusion coefficients of reacting species and (2) enhancing the thermodynamic favorabilities of the reactions for synthesizing TaB_2_ as well as converting intermediate TaC into TaB_2_.

9Ta_2_O_5_(s) + 11B_4_C(s) = 14TaB_2_(s) + 11TaC(s) + 15B_2_O_3_(l) (2)

7TaC(s) + 2B_4_C(s) + 3B_2_O_3_(l) = 7TaB_2_(s) + 9CO(g) (3)

**Figure 4:**
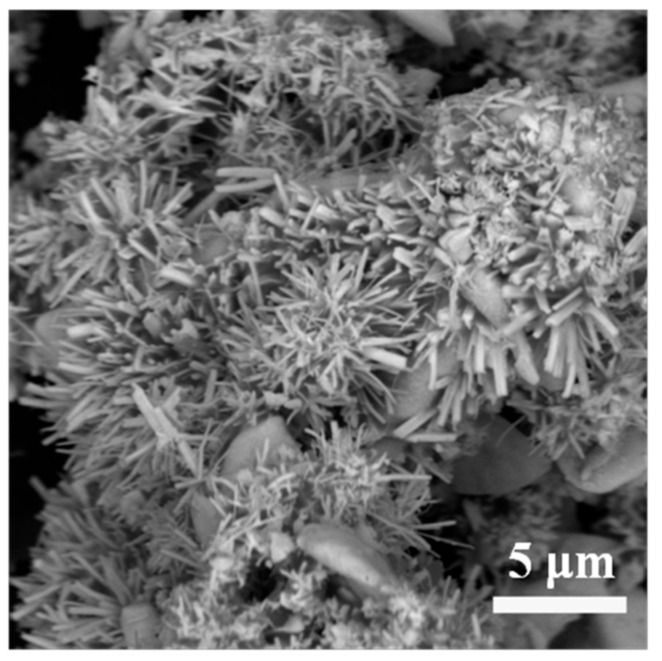
FE-SEM image of the morphology of the TaB_2_ powders (MSMBC-3) prepared by MSM-BCTR method at 1200 °C/20 min.

Microstructure and morphology of as-prepared phase pure TaB_2_ powders (MSMBC-3) were characterized. According to the FE-SEM observation (presented in Figure 4), similar to the TaB_2_ synthesized by Shah et al. [23], the particles in this sample exhibited the uniform morphology of urchin-like nanoflowers, which consist of numerous loosely agglomerated nanowires with average diameters and lengths of 410 nm and 4.16 μm, respectively, and a high aspect ratio (>10). Furthermore, the results of EDS-mapping analysis evidence that elements of Ta and B in an atomic ratio close to 2:1, were homogeneously distributed over the as-observed urchin-like nanoflowers, verifying that they were TaB_2_ and the as-synthesized TaB_2_ powders resulting from the MSM-BCTR were of high purity.

**Figure 5:**
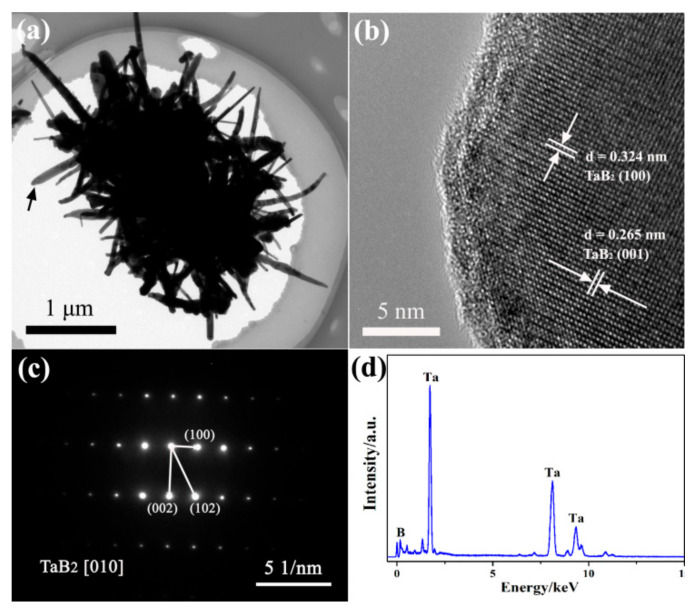
(**a**) Low-resolution TEM image, (**b**) high-resolution TEM image of a representative urchin-like TaB_2_ nanoflower in MSMBC-3, (**c**) SAED pattern, and (**d**) EDS of an independent TaB_2_ nanowire (marked by a black arrow in (**a**)).

For further revealing the microstructure of as-observed rod-like particles, TEM examination was also conducted. The HRTEM image (Figure 5b, taken in the area defined by a black-dotted circle in Figure 5a) of a well-defined nanowire in an urchin-like nanoflower, shows that a highly arranged atomic lattice and constant interplanar spacings, along the mutually perpendicular directions, of 0.324 nm and 0.265 nm, which matched well with the interplanar spacings of (100) and (001) crystal planes of TaB_2_ crystal, respectively. In addition, the SAED pattern (Figure 5c) further verifies that this representative nanowire has a single-crystalline structure, and its anisotropic morphology is formed by epitaxial growth along the [010] direction of the hexagon-system TaB_2_ crystal.

Therefore, it is reasonable to conclude that the TaB_2_ powders resulting from the MSM-BCTR method generally had a low extent of aggregation, large specific surface area, single-crystalline nature, and highly oriented anisotropic morphology, which were very favorable for enhancing not only sinterability but also the integral structural performance represented by fracture toughness of their bulk counterparts [24,25,26]. The achievement of low-temperature, highly efficiency preparation of such high-quality TaB_2_ powders can be mainly attributed to the combined effects of molten-salt medium and microwave heating.

Above all, compared to the conventional thermal reduction method, the MSM-BCTR method possessed versatile significant merits in the preparation of TaB_2_ and other raw material powder for UHTCs as well as TaB_2_, including the low material cost, ultra-high efficiency, low energy consumption, and the potential in optimizing sinterability as well as the overall mechanical properties (especially fracture toughness) of their bulk counterparts, which may shed substantial light on further development of the methodology for industrial production of UHTCs powders.

## 4. Conclusions

By taking advantage of the highly efficient and energy-saving approach of molten-salt and microwave comodified boro/carbothermal reduction, phase-pure TaB_2_ powders were successfully prepared by firing the less expensive raw powders of Ta_2_O_5_/B_4_C at a reduced temperature of 1200 °C for only 20 min, which were not only remarkably milder than those (firing temperature > 1250 °C and dwell time of dozens of hours) required by the conventional thermal reduction method using the same type of reducing agent, but also much more efficient (at least several hours) even than the methods using a vast amount of strong, but expensive, reducing agent of elemental boron. Moreover, the as-obtained TaB_2_ powders exhibited a single-crystalline structure, well-grown morphology of an urchin-like nanoflower, large specific surface area, low agglomeration extent of nanowires with high aspect ratio of >10, and great potential in improving sinterability and toughening their bulk counterparts. Such promising results were mainly attributable to the synergistic effects of molten-salt medium and microwave heating.

## Figures and Tables

**Table 1:** Batch compositions and processing parameters for synthesis of TaB_2_ by either MSM-assisted or conventional BCTR method.

Sample No.	Molar Ratio		Heating Mode	Temperature (°C)	Dwelling Time (min)	Salt Medium
	Ta_2_O_5_	B_4_C				
MSMBC-1	1.0	2.9	MWH ^a^	1000	20	NaCl/KCl
MSMBC-2	1.0	2.9	MWH	1100	20	NaCl/KCl
MSMBC-3	1.0	2.9	MWH	1200	20	NaCl/KCl
MSMBC-4	1.0	2.8	MWH	1200	20	NaCl/KCl
MSMBC-5	1.0	3.0	MWH	1200	20	NaCl/KCl
MSMBC-6	1.0	2.9	MWH	1200	20	—
MSMBC-7	1.0	2.9	CH ^b^	1200	20	NaCl/KCl

^a,b^ MWH and CH denote microwave heating and conventional heating processes, respectively.

## Data Availability

For anyone who is interested with the data of this article, please contact with corresponding author.
